# Supplementary material for: Structures of peptide-free and partially loaded MHC class I molecules reveal mechanisms of peptide selection
Source: Nat Commun. 2020 Mar 11;11:1314. doi: 10.1038/s41467-020-14862-4 (PMC7066147; doi:10.1038/s41467-020-14862-4)
Supplement: Supplementary file 1 — Supplementary Information [file 41467_2020_14862_MOESM1_ESM.pdf]

Supplementary Information

Structures of peptide-free and partially loaded MHC class I molecules reveal mechanisms of peptide selection

Raghavendra Anjanappa, Maria Garcia-Alai et al

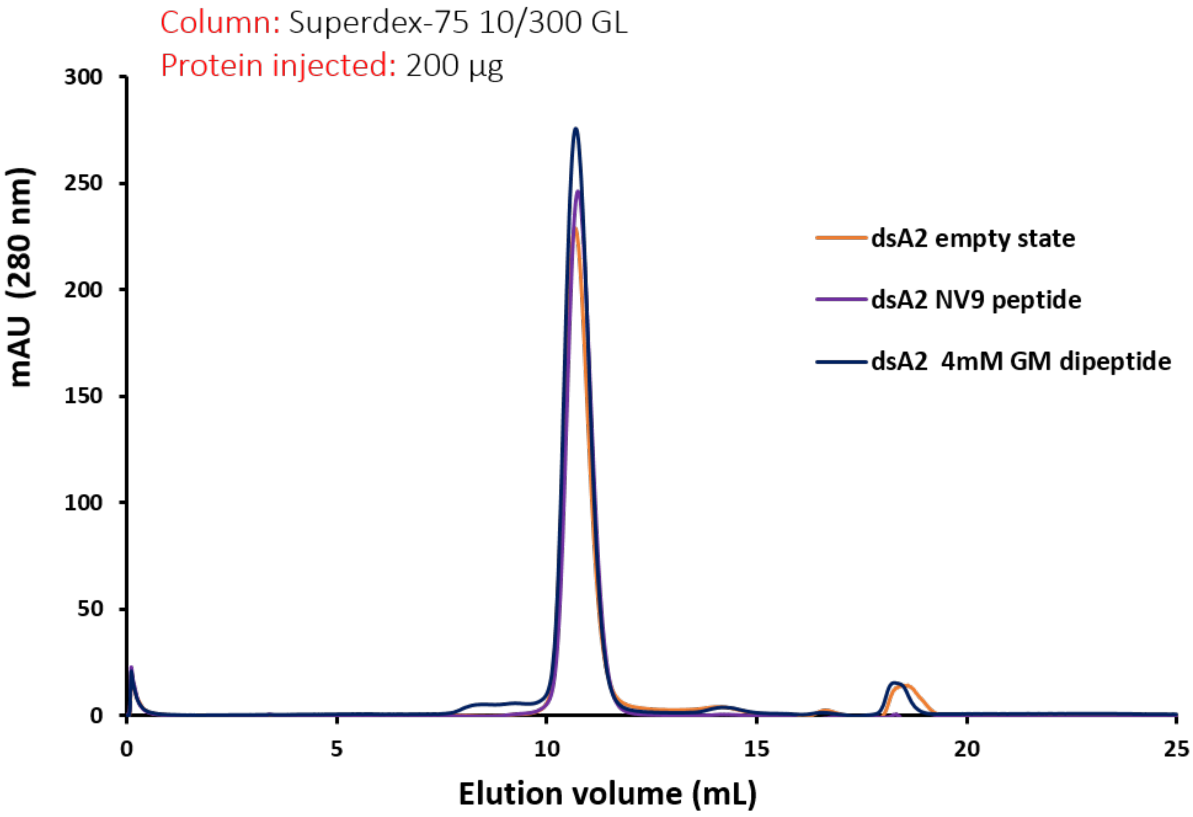

Supplementary Figure 1. Comparative stability profile of dsA2 protein molecules in the Size exclusion chromatography (SEC): Purification of folded dsA2 empty state (orange), and dsA2 NV9 peptide (purple) was performed without peptide in the running buffer and dsA2 GM dipeptide (Blue) purification in the presence of 4mM GM dipeptide in the running buffer.

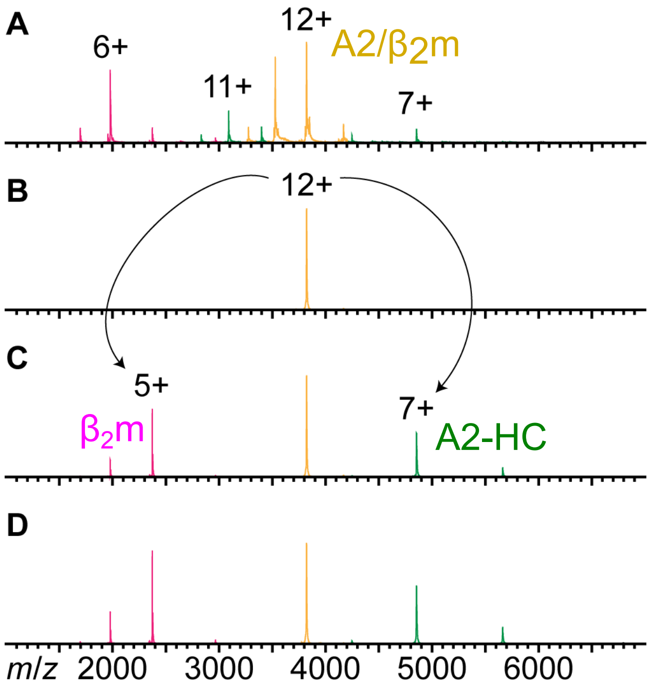

Supplementary Figure 2. MS/MS analysis of empty dsA2. A) Mass spectrum of the empty dsA2. **B)** The 12+ peak at 3820 *m*/*z* resulting from a native mass spectrum of dsA2 (yellow) was selected for MS/MS analysis at 50 V in the collision cell. **C)** Mass spectrum at a collision voltage of 75 V and **(D)** 100 V shows the dissociation of the complex, which allowed an accurate mass determination of the heavy chain (green) and β_2m (pink).

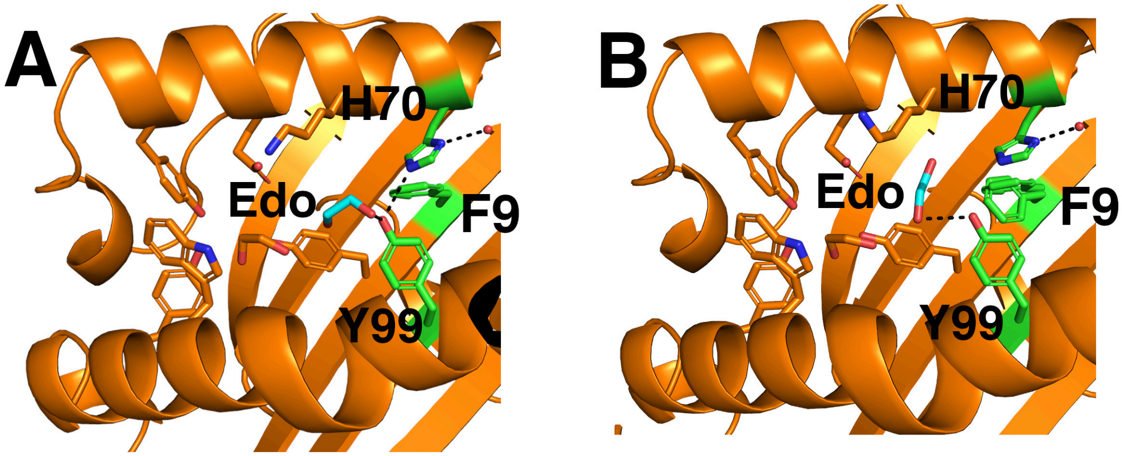

Supplementary Figure 3. Overview of the A pocket in the dsA2-peptide_free-2 structure achieved by high-salt treatment for both molecules in the asymmetric unit (in gold), compared with the dsA2-GM$_2$ structure (grey). A) In the #1 dsA2/peptide_free-2 molecule of the asymmetric unit, an EDO molecule sits in the area occupied by the main chain of P2 in a peptide-filled HLA-A2 structure. **B)** In the dsA2/peptide_free-2* molecule of the asymmetric unit, an EDO molecule sits in the area occupied by the side chain of residue P2 in a peptide-filled HLA-A2 structure, leading to conformational changes in residues Phe9 and His70.

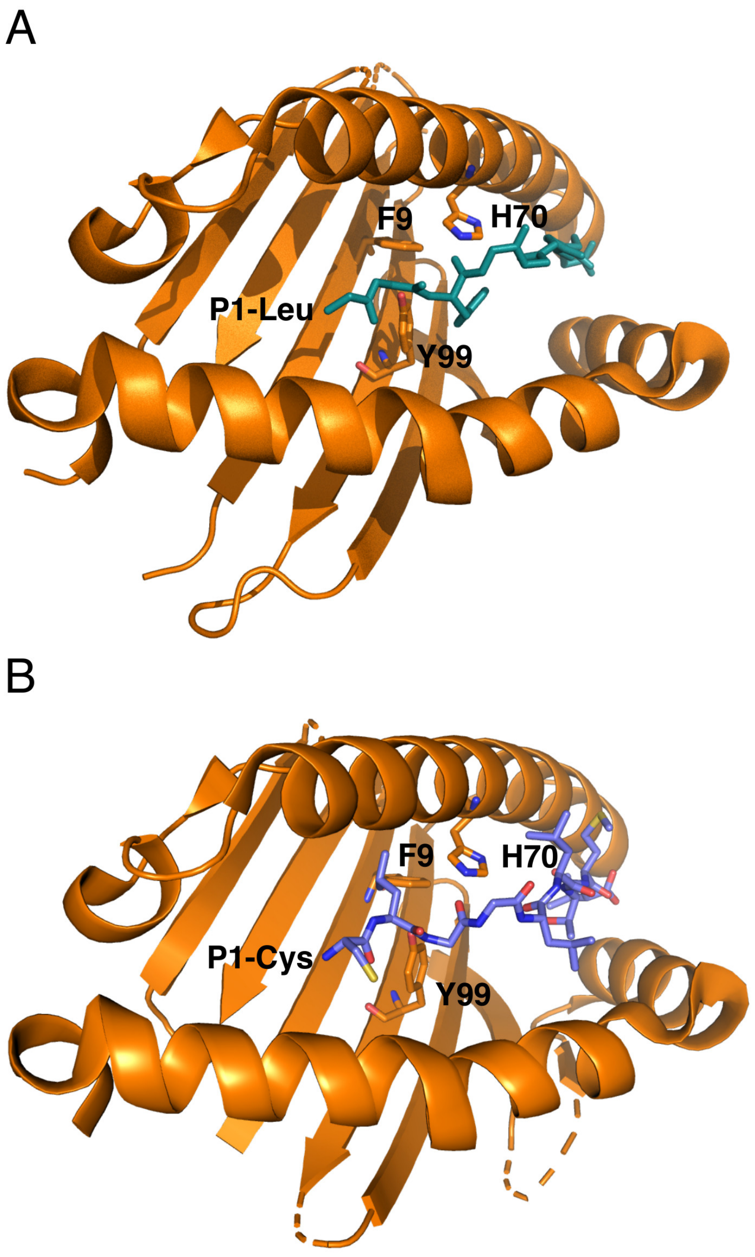

Supplementary Figure 4. The wtA2/LGYGFNVNY complex **(A)** (PDB 3PWL) contains a tyrosine at P3 that points down into the B pocket, sterically hindering the His74-Tyr99 interaction, thus opening the A pocket and creating a void that is filled by the side chain of Phe9[2]. The tyrosine side chain reaches towards Arg97, to close the F pocket. It was reported that wtA2/LGYGFNVNY is thermally unstable compared to the complex of wtA2 with the canonical peptide (LGYGFNVYI) (T_m, 40 °C *vs.* 60 °C)[3]. The HLA2/CLGGLLTMV complex **(B)** (PDB 3REW) contains a glycine in P3[4]. The lack of a side chain in this position also creates a void that is filled by the side chains of Phe9 and His70 of A2.

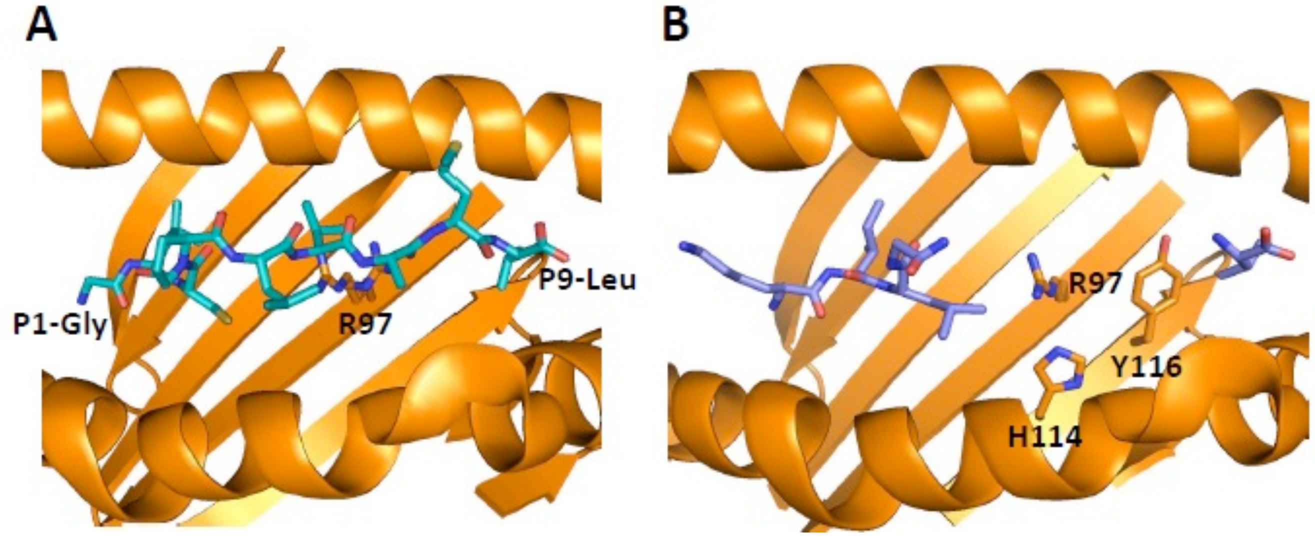

Supplementary Figure 5. (A) positioning of the side chain of Arg97 in this exceptional A2/GLCPLVAML structure (PDB 3MRF, unpublished) distorts the main chain conformation of the peptide in P6, leading to a violation in the dihedral angles according to the Ramachandran plot. The side chain of the valine residue in P6 is pushed upwards by the arginine side chain. **B)** Another illustration of the allosteric interactions amongst the Arg97/His114/Tyr116 triad can be observed in an A2 structure (PDB 2X4N) that contains the cleavage product of a UV light-exposed peptide, where there is still a tetrapeptide sticking out of the A pocket and a single amino acid in the F pocket[5]. Here, Arg97 is pointing towards the middle of the peptide binding groove to make contacts with the tetrapeptide cleavage product, whereas the Tyr116 is locking the F pocket around the remaining amino acid in the P9 position.

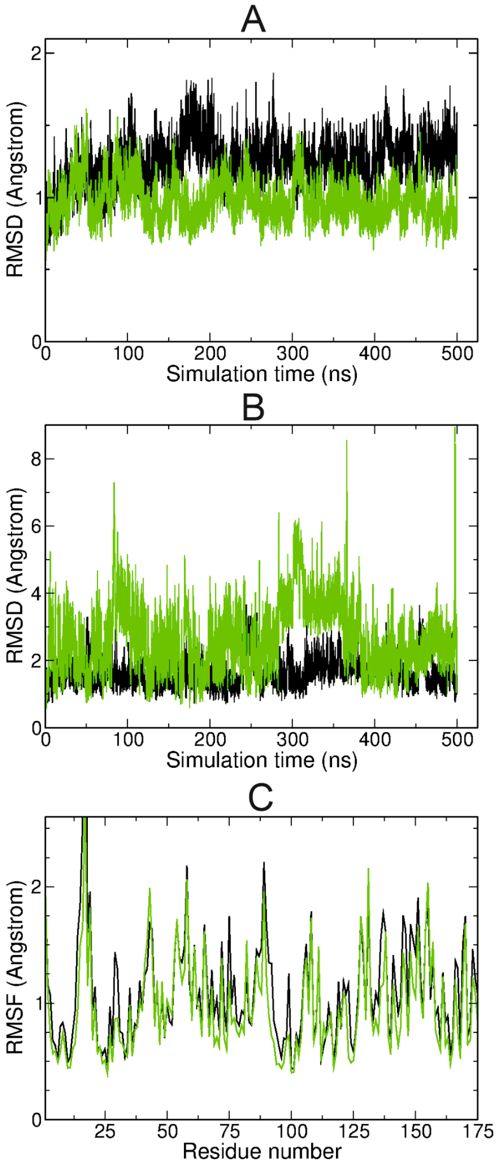

Supplementary Figure 6. A) Root-mean-square deviation (RMSD) of the protein backbone (residues 1-175, α1-α2-domain) from the start structure during MD simulations vs. simulation time of the empty dsA2 (black) and dsA2/GM_2 (green) structures. Start structures corresponded to the equilibrated crystal structures. The RMSD was calculated after best backbone superposition of residues 1-175 onto the corresponding crystal structure. **B)** RMSD (non-hydrogen atoms) of the GM dipeptide bound to the A pocket (black) and bound to the F-pocket (green) from the start structure vs. simulation time in the simulation of dsA2/GM_2. The RMSD of the dipeptides was calculated after best superposition of residues 1-175 onto the corresponding start structure. **C)** Root-mean-square fluctuation (RMSF) of residues 1-175 with respect to the mean structure (of residues 1-175) for the simulations of empty dsA2 (black) and dsA2/GM_2 (green).

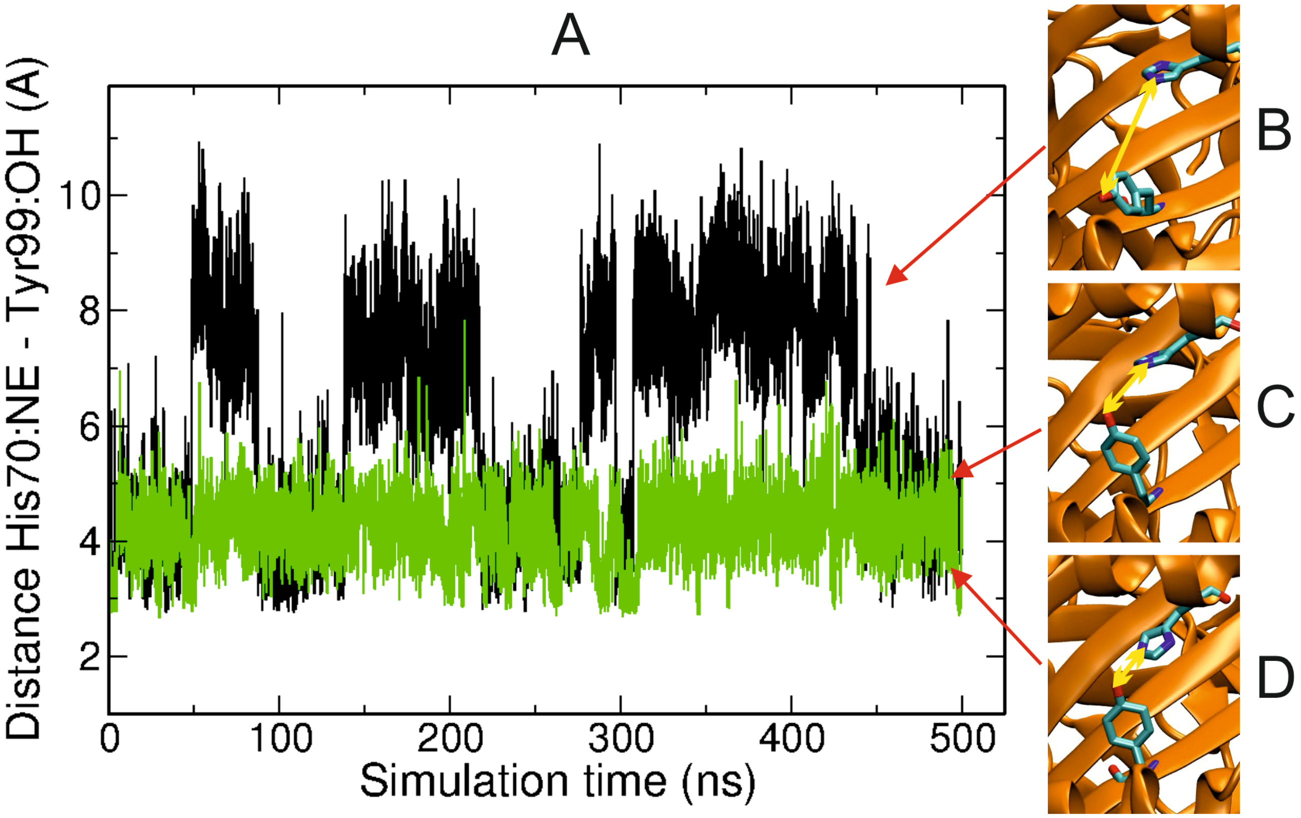

Supplementary Figure 7. A) Calculated distance between the Nε atom of residue His70 and the OH (Oxygen) of Tyr99 during MD simulations of empty dsA2 (black line) and dsA2/GM_2 (green line). The distance between His70 and Tyr99 characterizes the locked and unlocked states of the A pocket. Snapshots from the empty dsA2 simulation representing different His70-Tyr99 distances (indicated as red arrows in the plot) are shown in **B-D** (His70 and Tyr99 as stick models, yellow double arrow indicates the His70:Nε - Tyr99:OH distance in each snapshot). A short distance (hydrogen-bonded or near hydrogen-bonded) represents a locked state whereas large distances represent an unlocked state (disruption of His70/Tyr99 contact).

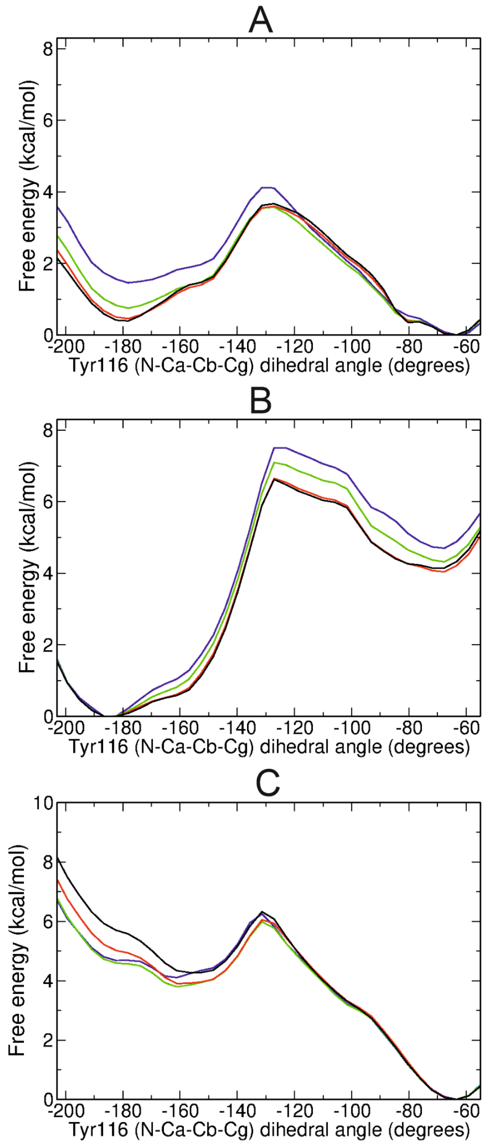

Supplementary Figure 8. Convergence of calculated potentials of mean force (PMF) of the Tyr116 side chain flip in the presence and absence of bound peptides. **A)** Cumulative calculated PMF for 20 ns (blue line), 30 ns (green line), 40 ns (red line) and 50 ns (black line) sampling per Umbrella Sampling (US) window for empty dsA2. **B)** same as (A) but for dsA2/NV9. **C)** same as (A) but for dsA2/GM_2.

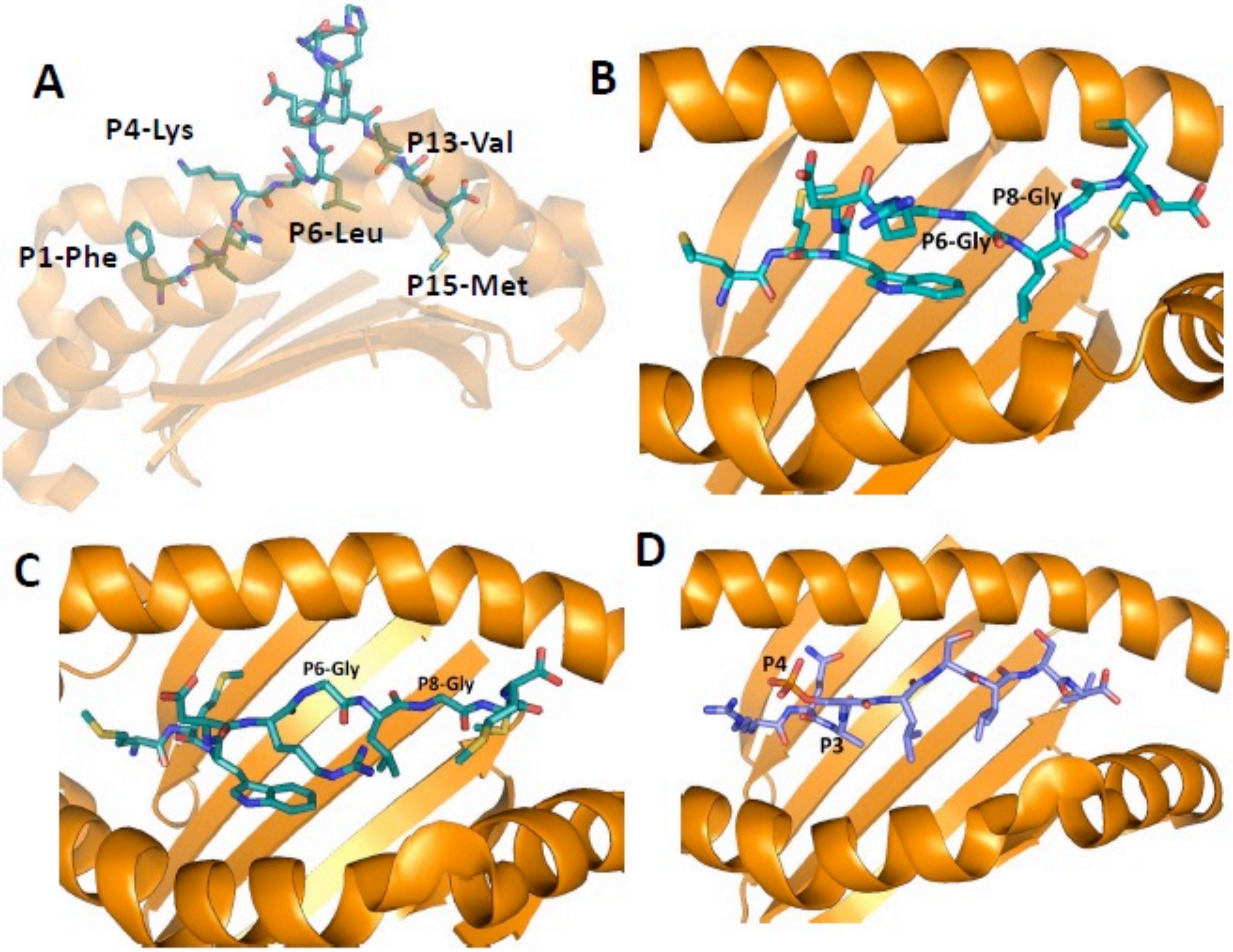

Supplementary Figure 9. wtA2 structures with a peptide containing methionine in the F pocket. A) contains a 15 amino acid peptide that loops out of the peptide binding groove between P6 (the sixth residue of the peptide) and the C-terminal methionine at P15 (PDB 4U6Y). Two unpublished structures **B) and C)** (PDB codes 6AMT and 6AMU) contain decamer peptides that have glycines in P6 and P8, and an additional anchoring tryptophan in P3, to improve binding. **D)** (PDB 3BGM) contains a phosphoserine peptide (RQAS*LSISV) at position P4, which compensates for the suboptimal anchor residues including methionine at P9. The non-phosphorylated peptide has a million-fold higher K_d (2 μM) and is not a high affinity binder for A2[1].

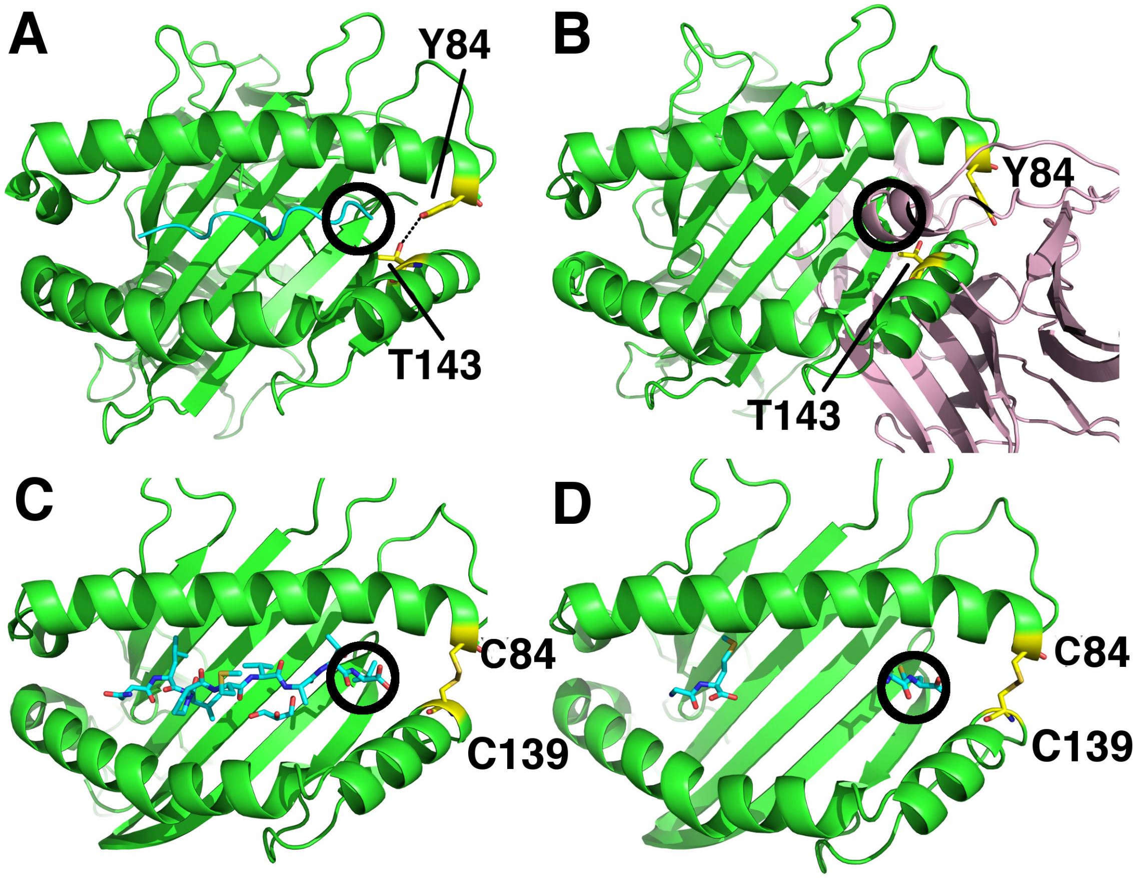

Supplementary Figure 10. Comparison of the murine TAPBPR/Db complex and the human dsA2 structures, showing the universal role of the F pocket in peptide loading. **A)** Ribbon diagram of the murine D^b structure in complex with an influenza peptide (PDB 1WBX). The F pocket is circled, and Tyr99 and Thr143 that delineate the border of the pocket are shown as sticks. **B)** The D^b/TAPBPR crystal structure, with the TAPBPR molecule shown in violet (PDB 5WER). **C)** the human dsA2/NV9 crystal structure, with the residues Y84C and A139C that form a designed disulfide bond shown as sticks. **D)** the dsA2/GM_2 structure showing the two dipeptides as sticks in cyan.

Supplementary Table 1. Native mass spectrometry

	mass species	*M*/Da	m_{exp}/Da (± SD/Da)	FWHM/Da (± SD/Da)
	β_2m - M1	11,731	11,729 ± 1	2.4 ± 0.5
	β_2m	11,862	11,860.0 ± 0.5	2.1 ± 0.4
wtA2	*heavy chain*	31,808	31,799 ± 4	7.3 ± 0.7
	A2 - M1	43,539	43,548 ± 5	6.3 ± 0.4
	A2	43,670	43,680 ± 2	6 ± 1
	A2 + adduct	44,009	44,017 ± 4	20 ± 10
dsA2	*heavy chain*	33,977	33,971 ± 2	7 ± 4
	A2 - M1	45,708	45,703 ± 2	4.1 ± 0.4
	A2	45,839	45,834 ± 3	4 ± 1
	A2 + adduct	46,178	46,172 ± 2	11 ± 7
	A2 + NLVPMVATV	46,782	46,778 ± 1	4 ± 1
	A2 + 2xNLVPMVATV	47,725	47,723 ± 3	8 ± 4

Experimental masses (m_{exp}) of the different protein species from wtA2 and dsA2 were determined from at least three independent mass spectrometry measurements. They are listed together with the respective values for standard deviation (SD) and average full width of the peak at half maximum (FWHM) along with the theoretically calculated molecular weight (*M*). FWHM values are given for the whole peak area where individual species were not fully resolved. β_2m-M1 and A2-M1 represent β_2m and A2 respectively, containing the N-terminal methionine.

Supplementary Table 2. Data collection and refinement statistics

Structure	dsA2/ peptide_free-1	dsA2/ peptide_free-2	dsA2/GM_2	dsA2/GM	dsA2/GL
Resolution range (Å)	84 – 1.75	85 – 1.70	84 – 1.60	81 – 1.65	81 – 1.40
Space group	P21	P21	P21	P21	P21
Major twinning fraction	0.81	0.63	0.90	None	None
Unit cell (a, b, c, β)	58.7, 82.7, 83.9, 90.1	58.6, 85.2, 83.9, 90.0	58.6, 84.1, 83.7, 90.0	52.9, 80.9, 56.3, 112.7	53.3, 81.1, 56.5, 112.3
Total reflections	205178	560550	353254	361759	290037
Unique reflections	76159	90573	103544	52645	86442
Multiplicity	2,7 (2.6)*	6.2 (6.3)*	3.4 (3.4)*	6.9 (2.6)*	3.4 (3.4)*
Completeness (%)	94.2 (96.2)*	100 (100)*	96.9 (95.3)*	99.9 (98.7)*	100 (99.3)*
Mean I/sigma(I)	7.3 (1.3)*	6.8 (1.9)*	9.7 (1.5)*	11.3 (1.6)*	14.2 (1.5)*
Wilson B factor	18.2	15.3	16.9	20.8	17.0
R-merge	0.095 (0.72)*	0.174 (1.00)*	0.069 (0.78)*	0.056 (0.62)*	0.030 (0.54)*
CC1/2	1.00 (0.57)*	0.99 (0.68)*	1.00 (0.68)*	0.99 (0.49)*	1.00 (0.64)*
Reflections used in refinement	72515	86445	98243	49987	82046
Reflections used for R-free	3641	4098	5274	2607	4366
R-work	0.177 (0.225)*	0.200 (0.230)*	0.169 (0.234)*	0.186 (0.250)*	0.190 (0.311)*
R-free	0.230 (0.350)*	0.248 (0.316)*	0.214 (0.312)*	0.224 (0.286)*	0.227 (0.310)*
Number of non-hydrogen atoms	7607	6591	3777	7718	3602
macromolecules	6442	6620	3243	6508	3118
ligands	23	51	12	37	13
solvent	1142	320	522	1173	471
Protein residues	750	750	375	758	375
RMS (bonds)	0.012	0.014	0.013	0.015	0.014
RMS (angles)	1.56	1.70	1.66	1.83	1.76
Ramachandran favored (%)	98.7	97.3	98.0	98.4	98.6
Ramachandran outliers (%)	0	0	0	0	0
Average B-factor	23.0	22.0	21.4	26.2	23.7
macromolecules	22.5	21.8	19.3	24.4	21.6
ligands	29.2	34.0	38.5	33.6	40.0
solvent	32.2	23.2	34.5	36.0	36.7

Supplementary Table 3. List of interactions between MHC residues and the dipeptide and ethylene glycol (EDO) molecules residing in the peptide-binding groove. Residues that form hydrogen bonds with the ligands are marked with *.

Molecule	Pocket	MHC residues
**dsA2/GM/GM**		
GM dipeptide	A	Met5, Tyr7*, Phe9, Phe33, Met45, Tyr59,Glu63*, Lys66*, His70, Tyr99*, Tyr159*, Trp167, Tyr171*
GM dipeptide	F	Thr73, Asp77*,Thr80, Leu81, Val95, Tyr116, Ala117, Tyr123, Ile124, Thr143*, Trp147*
**dsA2/GM/glycerol**		
GM dipeptide	A	Met5, Tyr7*, Phe9, Phe33, Met45, Tyr59,Glu63*, Lys66*, His70, Tyr99*, Tyr159*, Trp167, Tyr171*
Glycerol	F	Asp77, Thr80, Leu81, Tyr116, Tyr123, Thr143*, Lys146*, Trp147
**dsA2/GL/Glycerol**		
GL dipeptide	A	Met5, Tyr7*, Phe9, Phe33, Met45, Tyr59, Glu63*, Lys66, His70, Tyr99*, Tyr159*, Trp167, Tyr171*
Glycerol	F	Asp77, Thr80, Leu81, Tyr116, Tyr 123, Thr143*, Lys146*, Trp147
**dsA2/peptide_free-1**		
EDO	A	Met5, Tyr7*, Phe33, Tyr59, Glu63, Tyr159*, Thr163, Trp167, Tyr171*
EDO	F (2nd MHC molecule)	His74, Asp77*, Leu81, Val95, Tyr116, Tyr123, Trp147
**dsA2/peptide_free-2**		
EDO1	A	Met5, Tyr7*, Phe33, Tyr59, Glu63, Tyr159*, Thr163, Trp167, Tyr171*
EDO2	A	Tyr7, Glu63, Lys66, His70, Tyr99*, Tyr159
**dsA2/peptide_free-2, second molecule in the asymmetric unit**		
EDO1b	A	Met5, Tyr7*, Phe33, Tyr59, Glu63, Tyr159*, Thr163, Trp167, Tyr171*
EDO2b	A	Tyr7, Phe9, Met45, Glu63, Lys66, Val67, His70,Tyr99*, Tyr159

Supplementary references

(1) Mohammed, F.; Cobbold, M.; Zarling, A. L.; Salim, M.; Barrett-Wilt, G. A.; Shabanowitz, J.; Hunt, D. F.; Engelhard, V. H.; Willcox, B. E. Phosphorylation-Dependent Interaction between Antigenic Peptides and MHC Class I: A Molecular Basis for the Presentation of Transformed Self. *Nat. Immunol.* **2008**, *9* (11), 1236–1243. https://doi.org/10.1038/ni.1660.

(2) Borbulevych, O. Y.; Piepenbrink, K. H.; Baker, B. M. Conformational Melding Permits a Conserved Binding Geometry in TCR Recognition of Foreign and Self Molecular Mimics. *J Immunol* **2011**, *186* (5), 2950–2958. https://doi.org/10.4049/jimmunol.1003150.

(3) Borbulevych, O. Y.; Do, P.; Baker, B. M. Structures of Native and Affinity-Enhanced WT1 Epitopes Bound to HLA-A*0201: Implications for WT1-Based Cancer Therapeutics. *Mol. Immunol.* **2010**, *47* (15), 2519–2524. https://doi.org/10.1016/j.molimm.2010.06.005.

(4) Simpson, A. A.; Mohammed, F.; Salim, M.; Tranter, A.; Rickinson, A. B.; Stauss, H. J.; Moss, P. A. H.; Steven, N. M.; Willcox, B. E. Structural and Energetic Evidence for Highly Peptide-Specific Tumor Antigen Targeting via Allo-MHC Restriction. *PNAS* **2011**, *108* (52), 21176–21181. https://doi.org/10.1073/pnas.1108422109.

(5) Celie, P. H. N.; Toebes, M.; Rodenko, B.; Ovaa, H.; Perrakis, A.; Schumacher, T. N. M. UV-Induced Ligand Exchange in MHC Class I Protein Crystals. *J. Am. Chem. Soc.* **2009**, *131* (34), 12298–12304. https://doi.org/10.1021/ja9037559.
